# Carob: A Sustainable Opportunity for Metabolic Health

**DOI:** 10.3390/foods11142154

**Published:** 2022-07-20

**Authors:** Aristea Gioxari, Charalampia Amerikanou, Irini Nestoridi, Eleni Gourgari, Harris Pratsinis, Nick Kalogeropoulos, Nikolaos K. Andrikopoulos, Andriana C. Kaliora

**Affiliations:** 1 Department of Nutritional Science and Dietetics, School of Health Science, University of the Peloponnese, Antikalamos, 24100 Kalamata-Messinia, Greece; a.gioxari@uop.gr (A.G.); irininest@gmail.com (I.N.); elenigourgari@gmail.com (E.G.); 2 Department of Nutrition and Dietetics, School of Health Science and Education, Harokopio University, 70 El. Venizelou Ave, 17676 Athens, Greece; amerikanou@windowslive.com (C.A.); nickal@hua.gr (N.K.); nandrikop@hua.gr (N.K.A.); 3 Laboratory of Cell Proliferation & Ageing, Institute of Biosciences & Applications NCSR “Demokritos”, Patriarchou Grigoriou E’ & 27 Neapoleos str., Ag. Paraskevi, 15341 Athens, Greece; hprats@bio.demokritos.gr

**Keywords:** carob, sustainable food, obesity, metabolic disorders, dyslipidemia, hypertension, insulin resistance, diabetes

## Abstract

Carob (*Ceratonia siliqua* L.) is an evergreen tree that belongs to the *Leguminosae* family and grows in the arid and semi-arid regions of the Mediterranean basin. The carob tree is resistant to droughts and salinity, while its deep root systems allow CO_2_ to sink, mitigating global warming effects. Traditionally, carob has been used to produce animal feed, but for many years, it was excluded from the human diet. Nowadays, agricultural and industrial sectors exploit carob fruit, also referred to as carob pod, and its primary products (i.e., flour, powder and syrup) to develop a variety of foods and beverages. The nutritional composition varies depending on the carob part but also on genetic, cultivar, seasonal and environmental factors. Despite the high sugar content, the carob pod is rich in insoluble fiber and microconstituents including phenolic compounds, inositols (mainly d-pinitol) and vitamins. In the present review article, we aimed to (a) highlight the role of carob cultivation in addressing climate change challenges and the need for sustainability, and (b) summarize the effects of carob consumption on obesity and related metabolic disorders.

## 1. Introduction

The Mediterranean diet (MD), a minimally processed plant-based diet, has been long recognized for its beneficial health effects against non-communicable diseases (NCDs), including obesity and related cardiometabolic disorders [1]. Obesity prevalence is escalating worldwide, reaching epidemic proportions; in 2019, prevalence reached 17% of the total adult population in the European region [2], while it is expected to exceed 20% by 2025, putting more pressure on individuals and health care systems [3]. The burden of obesity is also expanding to the developing nations as their economic transition advances [4]. Furthermore, obesity, a main component of the metabolic syndrome (MetS), is associated with loss of disease-free years [5], increasing the risk of all-cause mortality and cardiovascular disease (CVD)-specific mortality [6]. Metabolically healthy obese individuals may show low risk of disease development, but over time, an inclination toward an unhealthy state is evident [7]. The pathophysiology of obesity is still unclear; it involves metabolic processes that are regulated by food intake, appetite and feedback systems under a genetic–epigenetic–environmental interaction complex between the brain, gastrointestinal system, adipose tissue and endocrine organs [8]. For instance, obesity induced meta-inflammation is considered as a key underlying stimuli for the development of cardiometabolic disorders [9]. Fat accumulation in the adipose tissue, mainly the white adipose tissue, triggers the production of acute phase proteins e.g., C-reactive protein (CRP), and pro-inflammatory cytokines such as tumor necrosis factor-alpha (TNF-alpha), interleukin (IL)-1 beta and IL-6, while leucocytes are infiltrated into the inflamed tissues [9]. At the same time, the overproduction of reactive oxygen species (ROS) is associated with nuclear factor-κB (NF-κB)-mediated inflammation in obesity [10].

There is accumulating evidence that adherence to MD improves blood lipid profile, protects from inflammation and oxidative stress, and it favorably alters the gut microbiota composition [8]. Additionally, a weight loss effect has been attributed to MD due to its low-energy-dense foods and high fiber content, as well as its regulating effects on appetite and satiety [8]. At the cellular level, MD is associated with the suppression of NF-κB-mediated inflammation and upregulation of the Keap1/Nrf2/ARE antioxidant pathway, increasing the production of endogenous antioxidant enzymes such as glutathione and superoxide dismutase [11]. These favorable health effects of MD are potentially attributed to the high content and the synergistic effect of phytochemicals, mainly phenolic compounds (e.g., quercetin, epigallocatechin-3-gallate), as well as dietary fiber, mono-unsaturated fatty acids (i.e., oleic acid), polyunsaturated fatty acids, sterols, vitamins, minerals and trace elements [1,12].

Apart from its health benefits, the MD has recently gained significant attention globally due its low environmental footprint [13]. As climate change is a threatening reality, and the global warming effect becomes more pronounced, changes in diet and a shift to more sustainable dietary patterns rich in bioactive components are more urgent than ever. Climate change should be addressed as a pandemic due to its effects on human health. In the last few years, many scientists have emphasized the need to address this challenge along with obesity and undernutrition, the leading courses of poor health globally, into a single Global Syndemic [14].

The Mediterranean dietary pattern has not resisted the invasion from the West, paralleling social change, industrialization and urbanization. Therefore, we now need to revive the diet itself, and its role in public health, and redirect our nutrition. In this context, the search for new potential MD compounds is worth studying. As such, carob, a less known species of the Mediterranean, although neglected for centuries, has gained a lot of popularity due its high nutritional value and ecological advantages. The carob tree, which belongs to the *Leguminosae* family (or *Fabaceae* family) of the Rosales order, has been widely cultivated since ancient times for its edible pods [15]. The carob tree is resistant to droughts and salinity, while its deep root systems allow good adaptation to the Mediterranean climate [15]. Traditionally, carob has been used to produce animal feed. During the last few years, however, the agricultural and industrial sectors have exploited carob and its primary products, i.e., flour, powder and syrup, to develop a variety of foods and beverages [16]. The nutritional composition varies depending on the carob part (e.g., pulp vs. seeds), but also on genetic, cultivar, seasonal and environmental factors [17]. Despite the high sugar content, most studies agree that carob is rich in insoluble fibers and microconstituents, including vitamins and phenolic compounds with well-known anti-hyperglycemic, antioxidant and anti-inflammatory properties [17,18,19,20].

Therefore, in the present review article, we aimed to: (a) highlight the role of carob and its cultivation as an integral part of the Mediterranean dietary pattern in addressing climate change challenges and the need for sustainability, and (b) summarize the effects of carob consumption on obesity and related metabolic disorders, i.e., CVDs, insulin resistance, type 2 diabetes mellitus (T2DM), non-alcoholic fatty liver disease (NAFLD), hypertension.

## 2. Carob; Composition in Nutrients and Non-Nutrient Bioactive Compounds

The carob tree grows in the arid and semi-arid Mediterranean regions, and it is native to the Middle East; the ancient Greeks brought it to Greece and Italy, while Arabs spread it across the North African coast, Portugal and Spain [15,21]. The official name of carob, “*Ceratonia siliqua* L.”, derives from the Greek word “keras” and the Latin word “siliqua” that describes the hard texture of the pod [21]. 

**Figure 1:**
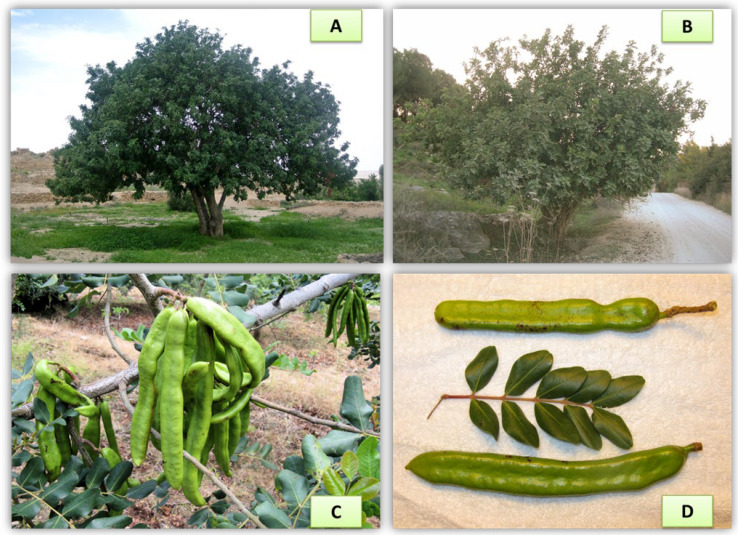
The carob tree (**A**,**B**) and the carob pod (**C**,**D**).

The carob tree is an evergreen dioecious shrub with a thick trunk and strong branches that grows up to 8–17 m (Figure 1A,B). The carob pod (Figure 1C,D) is an edible bean, commonly known as locust bean, which comprises pulp and the seeds (90% and 10%, respectively) [15,21]. The ripe pod is brownish and has an elongated and compressed shape (straight or curved) of varying dimensions with a wrinkled surface [15,21]. The pulp consists of a rough outer layer, the pericarp, and a soft inner layer, the mesocarp. A number of hard-textured and ovate-shaped brown-colored seeds (about 10 mm length, and 0.2 g per seed weight) are transversally located inside the pod separated by mesocarp. Carob seeds are obtained after the pods are broken, and they are composed of the coat (30–35%), the white and translucent endosperm (40–50%) and the germ or embryo (20–25%) [7,13]. Leaves are sclerophyllous covered by a very thick epidermis, which is considered to be rich in phenolic compounds [15,21]. Farmers rely on regular budding and grafting to improve fruit quality and yield, leading to the development of several local cultivars with different features over the centuries [22,23,24,25]. Yet, most cultivars today are of unknown origin with high genetic variation in morphologic and agronomic features [25,26]. To overcome this, identifying the associations between morphology, metabolome and genotype across the different agro-environments will enable carob to better capture its intrinsic physicochemical properties and stabilize them against environmental variations. 

The carob pod is rich in sugars (45–56% *w*/*w*) of which sucrose is up to 95% [27,28,29]. Glucose and fructose are found in lower concentrations, namely 2–4% and 6–7%, respectively [28]. Other carbohydrates such as maltose, raffinose, stachyose, verbascose, and xylose have been also identified in small proportions, but inositols are present in appreciable amounts [30]. Carob pods are an excellent source of other bioactive compounds that counteract the high sugar content, mainly dietary fiber, which accounts for up to 40% *w*/*w* of carob [17]. The insoluble fraction of carob fiber includes cellulose, hemicellulose and lignin, and it accounts for up to 70% of total carob fiber [31]. Soluble dietary fiber is present in small amounts (maximum 10 g × 100 g^−1^ carob fiber) [31]. The carob pod is high in phenolic compounds: mostly phenolic acids, gallotannins and flavonoids, and it is characterized by elevated antioxidant potential [32,33,34]. The content of polyphenols varies greatly depending on environmental and genetic factors as well as the extraction method followed; therefore, the total concentration ranges between 7.1 and 382.0 mg gallic acid equivalents per 100 g [19]. Phenolic compounds (such as gallic acid, gallotannins, cinnamic acid, myricetin) are also present in carob fiber at a concentration of 3.94 g/kg [35]. 

Furthermore, carob is low in fat (0.2–1.0%) [27,28,29] and contains appreciable amounts of protein, i.e., 2–7% [28], which is mostly present in seeds and particularly in the seed germ [36]. Depending on the harvest season, the total protein content and amino acid composition vary [37]. The carob pod contains significant amounts of minerals as well, including potassium (970–1089 mg/100 g), calcium (266–319 mg/100 g), phosphorous (76–79 mg/100 g) and magnesium (55–56 mg/100 g) [38,39]. 

Carob pods have received a lot of attention by the scientific community because of their high nutritional value and potential beneficial health outcomes. In recent years, carob has been increasingly used in food industries in order to develop innovative food products with functional properties: bakery and pastry products, carob-fortified foods, fermented and non-fermented pasta, carob-based milk beverages and water decoctions [16]. The primary carob products are flour, powder, and syrup, which are obtained from the carob pulp [40]. Initially, carob pods are dried to reduce moisture content to around 8%, and then, they are kibbled to separate seeds from the pulp portion [40]. Carob pulp, which comprises 90% of the carob fruit, contains a high percentage of sugar (48–56%), mostly sucrose (32–38%), glucose (5–6%) and fructose (5–7%), and a substantial amount of insoluble fiber (68.4%), phenolic compounds (gallic acid, gallotannins), minerals and antioxidant vitamins C and E [16,19,35]. However, it is poor in fat (0.2–0.6%) and protein as well (3%) [8]. Carob pulp is a cost-effective material for industrial food production as it contains sugar, fiber and antioxidants in their natural state [16].

Carob pulp can be kibbled to various grades to produce animal feed. For human consumption, carob pulp is dehulled, ground, roasted and finally milled to powder, which is commonly referred to as carob powder or flour [40,41]. Carob powder is broadly used as a chocolate or cocoa substitute that does not contain caffeine and theobromine [40]. It is worth pointing out that the total polyphenol concentration in carob powder is similar to that of cocoa (844 mg GAE/100 g vs. 929 mg GAE/100 g, respectively), highlighting the nutritional value of this carob product [42]. 

The production of carob syrup involves draining and boiling after leaving the grounded pulp in water [38]. Carob syrup does not contain oils; it is rich in sugars (63.88%), mainly sucrose, glucose and fructose [38], as well as bioactive d-pinitol (84.63 g/kg) [43]. Carob syrup contains considerable amounts of minerals, namely potassium, phosphorus, and calcium. It contains higher levels of silver and titanium than the carob fruit but no zinc [38]. Antioxidant vitamin C has been also identified in carob syrup at 0.07 g/L [16]. Furthermore, carob pulp syrup has been used as a carbon source to efficiently produce carotenoids from red yeast *Rhodosporidium toruloides* [44].

Carob seeds represent 10% of carob fruit. Because seeds have a significantly different sugar composition from carob pulp, they are less widely utilized by the food industry [45]. The major macronutrient is protein (18.6%) [16], while sugars (5.2%) and fat (2%) are also found in lower concentrations [46]. With regard to protein composition, albumin plus globulin, gliadin, soluble glutenin, and insoluble glutenin have been fractionated in carob seeds [46]. Several amino acids, such as arginine (27.8 g/100 g), alanine (17.0 g/100 g), and the essential amino acids lysine (15.0 g/100 g), isoleucine (8.6  ±  0.08 g/100 g) and valine (7.3  ±  0.06 g/100 g) have been also identified [20]. Furthermore, several fatty acids (FAs) have been detected in the lipid fraction of carob seeds, including oleic (45%), linoleic (32.4%), palmitic (16.6%) and stearic (4.7%) acids, while the ratio of saturated-to-unsaturated fatty acids is about 22:78 [20]. In the lipid fraction, γ-tocopherol and α-tocopherol are found in appreciable amounts (53.1% and 43.1%, respectively, in the tested tocopherol fraction) [20]. Four classes of phenolic compounds have been identified: (1) flavonols (quercetins and kaempferol derivatives), (2) flavanols (catechins and procyanidins), (3) gallic acid and derivatives, and (4) hydrolysable tannins [45]. Carob seeds are rich in phenolic content with high antioxidant potential and ability for scavenging radicals [20,45,47]. 

Seed endosperm is obtained after removal of the seed coat and the subsequent chemical or thermo-mechanical treatment of the seeds [17]. It is rich in the polysaccharide galactomannan (80–85%) of which 50–65% is mannose and 14–18% is galactose. The seed endosperm is primarily used by food industries for the production of locust bean gum (or E410), which is a natural polymer with stabilizing, thickening and flavoring properties [48]. In addition to galactomannans, the commercially available locust bean gum powder contains 5% protein, 0.5% fat, 1.0% crude fiber and traces of glucose, rhamnose, arabinose and xylose [49]. The seed germ (or embryo) flour containing the greatest amounts of protein (55–67%) is obtained as a by-product of the seed processing, and until now, its main use has been the production of animal feed [17,36]. The major protein fractions quantified are albumin and globulin (32%), and also glutelin (68%). No prolamins are detected, including gluten, which allows their use in the production of gluten-free products [50]. The carob germ has also 6.6% lipids, of which 21% are polar [36]. Seed germ flour has also appreciable proportions of monounsaturated fatty acids, particularly oleic acid (34.4% of total FA content) [40], which is well documented to protect against cardiometabolic risks [51]. In addition, the high concentration of seed germ flour in phenolic compounds [52] and phospholipids (11.8% *v*/*v*) might increase the antioxidant potential and consequently the stability and shelf-life of carob-based bakery products [53].

Finally yet importantly, data on seed coat composition are rather scarce. In a recent study, Lakkab and co-workers revealed that the seed coat is rich in phenolic compounds with high antioxidant activity [54].

## 3. A Sustainable Source for Human Nutrition in Both Developing and Developed Countries

The global warming effect becomes more pronounced as the emissions of greenhouse gases (GHG) continue to increase [55]. The main human-generated GHG is carbon dioxide (CO_2_). Carbon dioxide is absorbed by oceans, land plants and trees, and it is emitted to the atmosphere by natural processes. The rise of emitted CO_2_ (together with other GHG, e.g., methane, nitrous oxide, fluorinated gases) heats the planet up, causing more water to evaporate into the atmosphere, which in turn elevates temperature further. The Intergovernmental Panel on Climate Change (IPCC) estimates that the average surface temperature of the Earth is likely to increase by 1.8 to 4.0 °C by the end of the 21st century [56]. The sector of “Agriculture, Forestry and Other Land Use”, commonly known as AFOLU, accounts for one-quarter (≈10–12 gigatonnes of CO_2_ equivalent per year) of global GHG emissions, which is mainly due to deforestation and agricultural emissions from livestock, crops and nutrient management [57]. At the same time, agricultural subsidies by governments help to maintain these high-emission food production systems [58]. The developing countries are accountable for the majority of agriculture GHG emissions; nevertheless, high-income nations still release great amounts of GHG [59]. In 2018, global emissions due to agriculture (within the farm gate including related land use and land use change) totaled 9.3 billion tonnes of CO_2_ equivalent [59].

**Figure 2:**
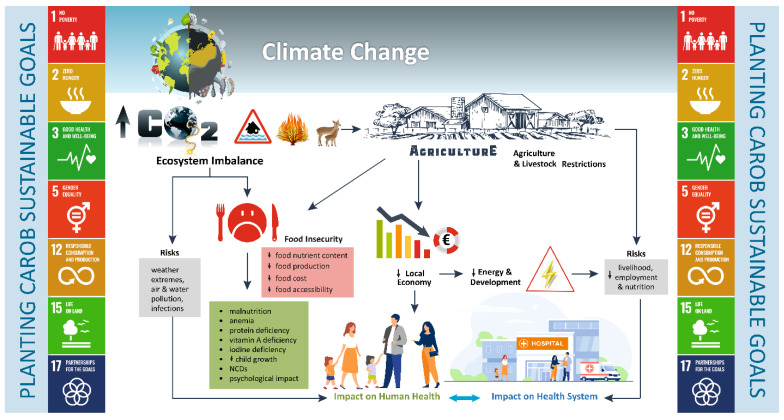
Interplay between climate change, food security, nutrition and human health. Planting carob trees might be a promising course of action to achieve the following sustainable development goals (SDGs) across the Mediterranean area: SDG1, to eradicate extreme poverty; SDG2, to end poverty; SDG3, to achieve good health and well-being; SDG5 to empower women in economic life; SDG12, for the responsible consumption and production; SDG15, for protecting, restoring and promoting the sustainable use of terrestrial ecosystems and reversing land degradation; and SDG17, for partnerships to achieve the goal.

Climate change and its aftermath, e.g., droughts, floods, storms, forest fires and extreme temperatures, have already brought about severe food crisis in the low-income countries, contributing substantially to the global hunger. Populations run the risk of infections, heat-related stress, respiratory diseases and shortages in drinking water and food, all contributing to poorer human health, income resources and livelihood, decreased productivity and employment, and finally migration [60]. The developed countries can easily address local food shortages resulting from weather extremes by importing foods [61]. However, the rising phenomena of climate change and the subsequent ecosystem imbalance will threaten food and nutrition security globally, hampering the productivity of crops, livestock, fisheries and forestry [60]. All other components of the food system are likely to be affected as well, i.e., storage, processing, distribution, food utilization and consumption. As a result, limited access to foods and a significant rise in food prices are potential threats, while the nutritional value of foods may decline [60,61,62,63]. Climate change is negatively correlated with food quality within the context of diversity, nutrient composition (macro- and micronutrients) and safety [64]. Crops such as wheat, potatoes, rice and beans, as well as fish and livestock, are most likely to be affected. The protein and lipid content of foods, as well as iron, zinc and vitamin A are expected to drop [62]. Consequently, individuals will be prone to develop malnutrition, anemias, protein deficiency, vitamin A deficiency, iodine deficiency, impaired child growth, non-communicable diseases (namely hypertension, hyperlipidemia, hyperglycemia, metabolic syndrome) and psychological disorders, placing more pressure on health systems [63,64,65,66,67,68,69]. According to the World Health Organization (WHO), climate change will result in approximately 250,000 additional deaths per year, mostly affecting the developing countries, low-income people and smallholder producers. The direct damage costs to health are estimated to lie between 2 and 4 billion US dollars per year by 2030 [69]. The impact of climate change on health is summarized in Figure 2. 

The Food and Agriculture Organization of the United Nations (FAO) highlights the role of sustainable land use in mitigating climate change and promoting food security and nutrition. Among the proposed measures is the cultivation of varieties resistant to flood, drought and/or saline [60]. Hence, planting carob trees might be a promising course of action to contribute to the sustainable development goals (SDGs) across the Mediterranean area: SDG1, to eradicate extreme poverty; SDG2, to end poverty; SDG3, to achieve good health and well-being; SDG5 to empower women in economic life; SDG12, for the responsible consumption and production; SDG15, for protecting, restoring and promoting the sustainable use of terrestrial ecosystems and reversing land degradation; and SDG17, for partnerships to achieve the goal. Carob shows resistance against droughts (xerophytic), and it has the ability to prevent erosion, soil degradation, and desertification [15]. According to Santos and co-workers (2019), regions with warm temperatures (16 to 36 °C) for more than half of the hours per year are favorable for the growth of carob trees [70]. Consequently, carob could play a significant role in the fire protection of agro-forest ecosystems in the Mediterranean countries [70]. Carob can be further used in reforestation actions to revalorize marginal lands, as CO_2_ sinks to mitigate global warming effects. It offers the advantage of growing in poor and unfertile soils of the Mediterranean and does not require much water for irrigation. Under the European Afforestation Scheme (1993–2008), afforestation with carob trees in Southern Spain covered more than 5000 hectares, mostly on low-quality agricultural land. In fact, the total carbon stock reached 41.75 Mg ha^−1^ and a total of 4091.5 Mg C for the whole plantation [71]. Similarly, Morocco has focused on planting carob trees to counteract deforestation [72].

During the last decade, there has been an effort to boost yield and develop a variety of carob-based foods with high nutritional value and potential health effects. According to FAOSTAT [73], the world production of carob in 2017 was 138,288 tons that derived from 38817 hectares mainly located in the wider Mediterranean area (60.9%) and Africa (19.1%). In particular, Portugal, Italy, Morocco, Turkey, Greece and Cyprus are the greatest carob producers. In 2017, production was higher from that of 2013 (114,823 tons; 42,218 hectares) and 2012 (126,405 tons, 42,713 hectares), although the area harvested was lower in 2017 [73]. These fluctuations could be partly attributed to the climatic and soil conditions during the crop cycle, as the majority of carob trees are not irrigated nor treated with mineral fertilizers [74]. Yield variations are also determined by endogenous factors related to alternate bearing practices [15]. Yet, one of the biggest concerns of the carob industry is the regional availability of pods, which must be consistent and regular year-round.

## 4. Effects on Metabolic Health

Online databases, i.e., Medline, Google Scholar, Scopus, Cohrane, and Web of Science were used for the literature search, inserting the following key terms: “carob, carob pod, carob fruit, carob seeds, obesity, body weight, glucose, insulin resistance, diabetes, cholesterol, blood lipids, hypertension, metabolic syndrome, fatty liver, liver steatosis”. The extracted articles fulfilled the following criteria: original research articles on humans or animals, written in English and published during the last 50 years (1972 to 2022). Three independent researchers were involved in literature searching and the selection of articles followed a hierarchical approach beginning from title, abstract and manuscript text. References from the selected articles were also used to complete the article review list. As a result, 47 original articles of preclinical and clinical studies were included (Tables S1 and S2, respectively).

### 4.1. Obesity, Meta-Inflammation and Dyslipidemia

Preclinical studies: Several preclinical studies (in vitro and in vivo) demonstrated the beneficial effects of carob and products on adiposity over the years. In 2018, Martínez-Villaluenga and coworkers investigated the effects of carob methanol extracts in 3T3-L1 mature adipocytes. Results showed that carob extract delipidized adipocytes and decreased triacylglycerol content (TG) by 32% [75]. Carob significantly reduced the production of potent inflammatory mediators, namely nitric oxide (NO) and prostaglandin D2 (PGD2). The same research group showed that extracts of carob pod, seed peel and germ were all efficient in reducing NO production by lipopolysacharide (LPS)-treated Raw 264.7 macrophage cells [76]. The authors conducted a 4-week experiment to investigate the effects of two different feeding carob formulations on 60 male Wistar rats with metabolic syndrome (MetS) [75]. Body weight, white adipose tissue weight, and interscapular brown adipose tissue weight did not differ significantly; only the blood non-esterified fatty acids did. The total amount of food intake or energy intake remained unchanged. The short intervention period (4 weeks) was considered a significant study limitation. However, inflammatory mediators, i.e., monocyte chemoattractant protein-1 (MCP-1) and interleukin-6 (IL-6), were significantly decreased in the group consuming carob versus control [75]. 

In 2017, Aboura and co-workers studied the effects of carob leaf infusions rich in phenolic compounds (i.e., 8.93 mg GAE/100 mL) in Swiss male mice fed on a normal diet (ND) or a high-fat diet (HFD) [77]. After six weeks, significant reductions were observed in body weight (BW) of animals fed on carob-HFD compared to HFD alone. As expected, the decrease in BW was less profound for the carob-ND group. The supplementation of HFD with carob leaf infusion caused a significant reduction in epididymal white adipose tissue weight. In line with this, the blood lipid profile, i.e., total cholesterol (T-CHOL) and triacylglycerol (TG) levels, significantly improved. In addition, tumor necrosis factor-alpha (TNF-alpha) and IL-6 in blood and organs (liver, spleen, adipose tissue and colon), major pro-inflammatory markers, significantly dropped compared to ND or HFD-controls. This anti-inflammatory property of carob leaves can be attributed to the inhibition of nuclear factor-kappa B (NF-κB) translocation in LPS-stimulated macrophages. Consequently, the production of nitric oxide, TNF-alpha and IL-6 were attenuated, leading to lower infiltration of macrophages into the adipose tissue [77]. 

Very recently, Fujita and co-workers confirmed the above results [78]; intake of carob pod polyphenols by male C57BL/6J mice was associated with reductions in BW, retroabdominal fat weight, fatty liver, liver TG levels, and adipocyte hypertrophy, as well as macrophage infiltration in the adipose tissue [78]. Adipocyte differentiation was suppressed by carob pod polyphenols through the post-transcriptional regulation of C/EBPβ, which is an important factor in mitotic clonal expansion during early differentiation, suggesting an anti-obesity effect. The beneficial effects of carob pod polyphenols were more pronounced after a shorter roasting time of carob pods (30 min versus 60 min) [78], which could imply that carob processing determines the beneficial properties of carob. 

Carob pulp exerts favorable health benefits against obesity as well; rats fed on a normal or a high-fat diet supplemented with 20% of carob pulp powder demonstrated significantly lower BW and adipose tissue weight compared to control [79]. These changes were accompanied by an improved blood lipid profile namely TG, total-cholesterol (T-CHOL), very low-density lipoprotein (VLDL) and low-density lipoprotein (LDL). Compared with control, the carob pulp group had significantly lower lipid peroxidation and higher plasma total antioxidant status as well as enhanced catalase (CAT) activity. Accordingly, feeding rats with 10 and 20% carob powder improved the lipid profile and histopathological characteristics in the hearts and kidneys of experimental rats [80]. El Rabey, Al-Seeni and coworkers observed similar hypo-lipidemic [81] and antioxidant activities [82] of methanolic carob powder extracts in hypercholesterolemic rats, although no effects were evident for BW. Additionally, significant reductions in liver weight, liver cellular damage, and serum liver enzymes such as alanine aminotransferase (ALT), aspartate aminotransferase (AST) and alkaline phosphatase (ALP) were detected, suggesting a substantial protection against liver steatosis [81]. The hepatoprotective properties of carob were in line with the enhanced activity of liver antioxidant enzymes, i.e., CAT, glutathione S-transferase (GST), glutathione peroxidase (GPx), superoxide dismutase (SOD), as well as lower liver oxidation [83,84]. Similar hypo-lipidemic, antioxidant, and anti-inflammatory effects were detected for a blend of carob pods and seed extracts together with fructo-oligosaccharides in rats with MetS [85]. 

The favorable effects of carob fiber rich in polyphenols on liver lipid metabolism may be attributed to its insoluble fraction [86]. As shown by Valero-Muñoz and co-workers (2017), the supplementation of carob fiber in the diet of dyslipidemic rabbits increased liver expression of sirtuin 1 (SIRT1) and peroxisome proliferator-activated receptor gamma coactivator-1alpha (PGC-1α), which are key modulators of the hepatic cholesterol and triglyceride metabolism [86]. The observation regarding the lipid-lowering effects of carob fiber dates back to 1979, when Würsch demonstrated that carob fiber prevents body weight gain in rats fed on high-cholesterol diet [87].

The hypo-lipidemic properties of carob fiber are also evident at the post-prandial state [88]. Carob fiber has been associated with decreased postprandial hypertriglyceridemia and cholesterolemia, as well as increased fecal moisture, fecal TG and CHOL [88]. In line with this, Pérez-Olleros and co-workers in 1999 observed a significant increase in fecal volume together with a decrease in blood T-CHOL (by 58%) and dietary fat absorption [89]. Sterol binding is more profound in the small intestine due to the high content in insoluble fibers. Additionally, the high content of non-fermentable polyphenols prevents the re-absorption of sterols trapped in the small intestine [89]. Further on, carob honey extract has been found to possess hypo-lipidemic and hepatoprotective properties, reducing blood lipids and liver enzymes [90]. The proposed mechanism underlying the observed hepatoprotection can be (a) attenuation of lipid peroxidation in the liver and (b) restoration of antioxidant enzyme activities [91,92].

Human trials: The evidence that the consumption of carob-based foods is negatively associated with hunger and energy intake from foods stems from a randomized, single-blinded and crossover trial in 50 healthy, non-diabetic and normal-weight men [93]. Participants were randomized to receive either 40 g of a carob snack or 40 g of a chocolate cookie (reference snack) following a standardized breakfast on an overnight fast [93]. The energy and macronutrient content of the two items were similar, but the carob snack was higher in dietary fiber (14.5 g/100 g) and lower in sugars (24.0 g/100 g) than the chocolate cookie (4.9 g/100 g and 36.4 g/100 g, respectively). Results showed that total amount of consumed food as well as total carbohydrate intake were significantly decreased 24 h after carob consumption compared to the reference food. Accordingly, consumption of the carob snack was associated with greater fullness and less perceived hunger, desire to eat, preoccupation with thoughts of food, and motivation to eat [93]. 

Carob seems to exhibit lipid-lowering effects as well. In an 8-week quasi-experimental study [94], 40 obese men were randomly assigned into four groups: (i) resistance training, (ii) carob supplementation, 1.5 g of carob seed powder in capsules per day (3 × 500 mg/d), (iii) combined i and ii, and (v) control group. Results showed that the combination of carob supplementation and resistance training significantly improved blood lipid profile and irisin index, which is an established adipokine released by the white subcutaneous adipose tissue [94]. 

In a clinical trial by Zunft and co-workers (2001), 47 adult volunteers with hypercholesterolemia received 15 g of a preparation from carob pulp rich in fiber on a daily basis for 8 weeks [95]. This carob preparation was added to three different products (breakfast cereal, fruit muesli bar, and powdered drink), each containing 5 g of carob, which were consumed regularly. Blood T-CHOL and LDL levels showed significant abatement, while TG and HDL remained unchanged. In another randomized, double-blind placebo-controlled trial in 58 adult hypercholesterolemic patients, participants consumed two servings of bread and one serving of fruit bar daily either with or without a carob pulp preparation (total 15 g/d) for 6 weeks [96]. Energy and macronutrient intake as well as anthropometric values such as BMI and hip circumference showed no significant change throughout the trial. However, the consumption of carob fiber reduced blood levels of T-CHOL, TG and LDL. The increase in apolipoprotein B:A-1 ratio in the control arm was attenuated in the intervention group [96].

Likewise, the results from a randomized, double-blind placebo-controlled trial of 2010 in 97 hypercholesterolemic patients showed that consumption of 8 g of carob pulp rich in fiber for 4 weeks was efficient in limiting the serum T-CHOL, TG, LDL and LDL:HDL ratio [97].

### 4.2. Effects on Glycaemia and T2DM

Preclinical trials: The high-fiber content of carob might be helpful in regulating glucose response in patients with T2DM. In 1989, Forestieri and co-workers were the first to observe carob’s effects on glucose and insulin modulation together with a BW and T-CHOL-lowering action [98]. In rats exposed to fiber-rich carob fruit extract following oral glucose loading, the area under the curve (AUC) of blood glucose significantly decreased postprandially [99]. After one week of the extract administration, AUC decreased further. The inhibition of α-glucosidase activity and sodium-glucose-linked 34 transporter-1 (SGLT1) were proposed as the potential underlying mechanisms of carob’s glucose-lowering effect [99]. When examining the effects of a carob fruit extract-enriched meat on rats with T2DM, the Homeostatic Model Assessment for Insulin Resistance (HOMA-IR) was significantly reduced, which was apparently modulated by the InsR/PI3K/AKT/GSK3 insulin-signaling pathway [100]. This carob fruit extract-enriched meat was associated with lower glycaemia and higher blood insulin levels as well as favorable changes in pancreatic beta cells and liver glycogen storage and steatosis [101]. Furthermore, some beneficial effects on gut microbiota, an increase in short-chain fatty acid production in the colon, and an improvement of colonic barrier integrity have been observed [102]. The favorable effects of carob’s fruit extract-enriched meat on glycemia and insulinemia were accompanied by significant ameliorations in growth rate, blood lipid profile, steatosis and liver oxidation as well [103]. 

An improvement of glycaemia and pancreatic function was also evident in diabetic rats receiving unripe carob pod extracts that were found to possess higher phenolic content (including flavonoids) than their mature counterparts did as well as enhanced radical scavenging activity on a dose-dependent manner [104,105]. Finally, the administration of carob-derived d-pinitol has been also associated with ameliorated glycemia due to the modulation of (a) C4A complement protein involved in the insulin secretion pathway, (b) insulin receptor-signaling pathway and (c) plasma ghrelin regulation [106,107]. 

A recent report regarding the ability of *C. siliqua* leaves’ methanolic extract to suppress glucose-mediated protein glycation leading among others to lower levels of advanced glycation end products (AGEs) is also very promising regarding the prevention of diabetic complications induced by high AGEs [108]. The high antioxidant activities of carob pods (pulp and seeds), as well as carob leaves in human cell lines, potentially make these products ideal for their incorporation into foods with benefits for health [109,110]. 

Human trials: To date, there have been few clinical trials investigating the effects of carob on blood glucose. In 2018, Papakonstantinou and co-workers showed that the consumption of 130 g of bread containing 10% of carob-seed flour (equivalent to 50 g available carbohydrates) reduced postprandial blood glucose at 15 and at 30 min compared to the reference food (D-glucose) in 10 healthy individuals [111]. Peak glucose value and 0–120 min glucose iAUC were significantly lower than the other tested breads (white, coarse bran and fine bran) of the same carbohydrate content. In another randomized crossover study in 20 healthy subjects (aged 22–62 years), plasma glucose, plasma total and acylated ghrelin, and serum insulin were accessed before and after (i) oral glucose load or (ii) carob fiber in a water–glucose solution over a period of 180 min [112]. Interestingly, intake of 5 and 10 g of carob fiber increased plasma glucose and serum insulin compared to control. The consumption of 20 g carob fiber did not result in a significant increase in plasma glucose compared to control. Plasma acylated ghrelin concentrations did not change, while total ghrelin decreased significantly after the consumption of 10 g of carob fiber. These results indicate that carob fiber administered with a water–glucose solution increases postprandial glucose and insulin responses, suggesting a deterioration in glycemic control [112]. Similar results were obtained by the same authors when embedding carob fiber in foods [113]. In the study of Santos and coworkers, it was found that carob tablets and carob flour have low glycemic indexes and low glycemic loads due to their high fiber content and particularly their insoluble fibers [114].

On the other hand, d-pinitol derived from carob seems to possess favorable effects on glycemic response both in healthy and diabetic individuals by decreasing inflammation, reactive oxygen species production, as well as by improving endothelial function [106,115,116,117]. Importantly, many other health-promoting effects of d-pinitol and its derivatives have led to its designation as a natural super-food [118].

### 4.3. Other Metabolic Disorders

As mentioned above, some studies on the effect of carob on obesity and diabetes have also shown a reduction in fatty liver or an improvement in steatosis [78,81,83,84,86,90,91,92,101,103] in both mice and humans. In addition, in 2019, Rico and his colleagues verified carob’s protective effects against non-alcoholic fatty liver disease. The consumption of carob-based snacks by NAFLD rats was associated with lower liver TG content due to an increase in fat b-oxidation and modulation of oxidative stress [119]. 

With regard to carob’s effects on hypertension, data are rather scarce. As shown in Supplementary Table S1, only two studies showed that carob was efficient in reducing angiotensin-converting enzyme (AE) activity [75,76], while one study demonstrated increased vascular function and improved arterial pressure [85]. Notably, honey produced by bees utilizing carob flowers has been shown to possess diuretic properties for experimental animals without causing them hypokalemia [120].

## 5. Conclusions and Perspectives

Carob, a well-known and neglected legume of the Mediterranean basin, is traditionally used for the production of animal feed. Nowadays, the evidence supporting its use in the human diet is rising. Its high content in insoluble fibers, phenolic compounds and inositols together with its low environmental footprint make carobs an ideal fit to the sustainable Mediterranean dietary pattern. Its low environmental footprint allows larger-scale cultivation even in areas with long periods of warm temperatures and droughts, reinforcing food security and the local economy as well. Planting carob trees might be a promising way to achieve some important sustainable development goals set by the FAO across the Mediterranean area.

**Figure 3:**
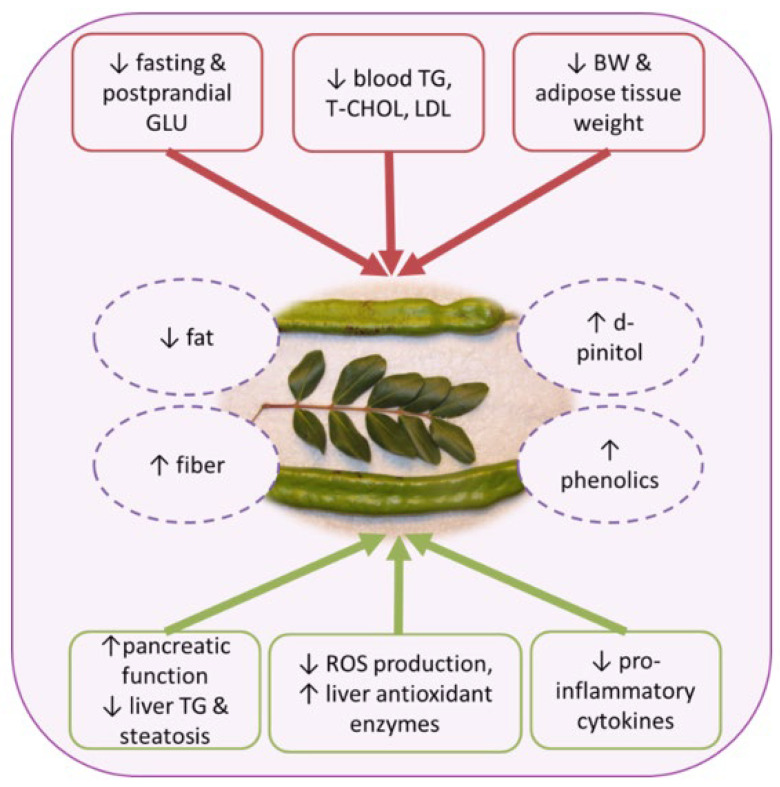
Effects of carobs on metabolic health. GLU, glucose; TG, triacylglycerols; T-CHOL, total cholesterol; LDL, low-density lipoprotein; ROS, reactive oxygen species; BW, body weight.

The majority of the reviewed research herein supports the contribution of carobs in metabolic health (Figure 3). In particular, significant ameliorations of body weight and adiposity, along with improved blood lipid profile, liver steatosis, glycemia and insulinemia has been described. Although data on humans are scarce and the effects on adiposity are controversial, carob consumption exhibits beneficial effects on blood lipids and postprandial blood glucose levels. To this point, a plethora of factors such as the genetic background as well as differences in the study design may influence the size effect. Additionally, sample size seems to be a major limitation; a great proportion of the reviewed trials were conducted on insufficient patient numbers, leading to a low power of analysis. Furthermore, very few clinical studies accessed lifestyle factors such as dietary and physical activity patterns that could affect the study outcome. Large-scale and well-designed randomized placebo-controlled trials are needed to confirm the potential effect of carobs on obesity (including weight loss) and related cardiometabolic disorders.

## Data Availability

The data presented in this study are available on request from the corresponding author.

## Supplementary Materials

The following supporting information can be downloaded at: https://www.mdpi.com/article/10.3390/foods11142154/s1, Table S1: Included preclinical trials investigating the effects of Carob (*Ceratonia siliqua* L.) on obesity and related metabolic disorders; Table S2: Included clinical trials investigating the effects of Carob (*Ceratonia siliqua* L.) on obesity and related metabolic disorders.
