# Supplementary material for: Carob: A Sustainable Opportunity for Metabolic Health
Source: Foods. 2022 Jul 20;11(14):2154. doi: 10.3390/foods11142154 (PMC9325207; doi:10.3390/foods11142154)
Supplement: Supplementary file 1 [file foods-11-02154-s001.zip › foods-1810514-supplementary.pdf]

Carob: A Sustainable Opportunity for Metabolic Health

Supplementary material

Supplementary Table S1. Included preclinical trials investigating the effects of Carob (*Ceratonia siliqua* L.) on obesity and related metabolic disorders

a/a	Ref. No	Daily Dosage	Design	Biomarker	Effect
1	[75]	✓ In vitro: carob pod methanol extracts at 50 or 100 μg/mL<br>✓ In vivo: animal feeding formulations of carob-seaweed-flour containing carob at 5.29 % or 4.49 %	✓ In vitro: 3T3-L1 mature adipocytes treated with carob pod extracts or ethanol<br>✓ In vivo: 60 male Wistar RccHan rats with MetS, 6 weeks old, 203 ± 2.5 g, 4 weeks study duration	In vitro:<br>✓ TG content, ACE activity, PGD2, NO, TNF-a<br>In vivo:<br>✓ BW, white adipose tissue weight, interscapular brown adipose tissue weight, gastrocnemius muscle weight, food intake, energy intake<br>✓ non-esterified fatty acids, water intake, SBP, MCP-1, IL-6<br>✓ circulating insulin<br>✓ blood GLU	↓<br>↔<br>↓<br>↑<br>↔
2	[76]	methanolic carob pod, seed peel and germ extracts	✓ In vitro: 3T3-L1 mature adipocytes and (LPS)-treated Raw 264.7 macrophage cells	✓ TG content, ACE activity, NO, PGD2, TNF-alpha	↓
3	[77]	✓ In vivo: a normal diet (ND) supplemented with polyphenol-rich carob leaf infusion 1%; a high-fat (HFD) diet supplemented with polyphenol-rich carob leaf infusion 1%<br>✓ In vitro: aqueous extracts of carob leaves at 10, 20 and 30 μg/mL	✓ In vitro: LPS-stimulated 264.7 murine macrophages<br>✓ In vivo: 50 Swiss male mice, 40 - 50 g, divided into groups of ND or HFD supplemented with carob leaf infusions (with or without DSS) vs. controls, 6 weeks study duration	In vitro:<br>✓ NO, TNF-alpha, IL-6, p65 NF-kappaB activation<br>In vivo:<br>✓ BW, epididymal white adipose tissue weight, blood GLU, insulin, TG, T-CHOL, TNF-alpha, IL-6, as well as tissue TNF-α, IL-6 and IL-1beta of all organs tested	↓<br>↓
4	[78]	carob pod polyphenols (CCP) from:<br>• 30 min roasting time, called “Light” (30.9 ± 1.8% of polyphenols), and<br>• 60 min roasting time, called “Extra Dark” (28.0 ± 1.5% of polyphenols)	✓ In vitro: 3T3-L1 preadipocytes<br>✓ In vivo: 36 male C57BL/6J mice, 8 weeks old, fed on a high-fat diet divided into 0.06% CCP-Light, 0.3% CCP-Light, 0.06% CCP-Extra Dark, 0.3% CCP-Extra Dark vs. controls, 52 days study duration	In vitro:<br>✓ TG accumulation, GPDH activity, mitotic clonal expansion, expression of transcription factors involved in adipocyte differentiation<br>In vivo:<br>✓ BW, retroabdominal fat weight, fatty liver, liver TG, adipocyte hypertrophy, macrophage infiltration in adipose tissue<br>✓ epididymal fat weight, T-CHOL	↓<br>↓<br>↔
5	[79]	20 % carob pulp powder	In vivo: adult male Wistar rats, 120 - 150 g, assigned to standard diet (control, N=5), standard diet supplemented with 20% carob pulp powder (N=5), fat-rich hypercaloric diet (N=5), fat-rich hypercaloric diet supplemented with 20% carob pulp powder (N=5), 2 months study duration	✓ BW, adipose tissue weight, blood GLU, TG, T-CHOL, VLDL, LDL, TBARS, LOOH<br>✓ ORAC, CAT activity<br>✓ HDL, vitamin C, carbonyl proteins	↓<br>↑<br>↔

6	[80]	carob powder 10 and 20 %	In vivo: 40 male Sprague–Dawley rats were divided into four groups (N=10/group) fed on: (1) a basal diet, (2) hyperlipidemic diet, (3) hyperlipidemic diet plus 10 % or (4) 20% carob powder, 6 weeks study duration	✓ TG, T-CHOL, VLDL, LDL, heart and kidney histopathological damage<br>✓ HDL<br>✓ BW	↓<br>↑<br>↔
7	[81]	20 % w/w of methanolic carob powder extract	In vivo: 24 male rats (Rattus norvegicus, 150 - 200 g, divided into the negative control group of fat rich diet (N=6), the hypercholesterolemic positive control of 2% cholesterol in the fat rich diet, hypercholesterolemic group of 2% cholesterol in the fat rich diet plus carob extract, 8 weeks study duration	✓ Liver weight, serum ALT, AST, ALP, T-CHOL, TG, LDL, VLDL, lactate dehydrogenase<br>✓ BW gain, food efficiency ratio, serum HDL	↓<br>↔
8	[82]	20 % w/w of methanolic carob powder extract	In vivo: 24 male rats (Rattus norvegicus, 150 - 200 g, divided into the negative control group of fat rich diet (N=6), the hypercholesterolemic positive control of 2 % cholesterol in the fat rich diet 9 (N=6), hypercholesterolemic group of 2 % cholesterol in the fat rich diet plus carob extract (N=6), 8 weeks study duration	✓ serum uric acid, creatinine, MDA<br>✓ blood total bilirubin, albumin to globulin ratio, urea, Na, K<br>✓ serum, liver and heart antioxidant enzymes: CAT, SOD, GSH, and GSR	↓<br>↔<br>↑
9	[83]	powdered carob pod adjusted to 10 % of the rat food	In vivo: 24 Wistar albino rats, 200 - 300 g, randomly divided into 4 groups (N=6/group) of (1) healthy control, (2) carbon tetrachloride (CCl_4)-induced hepatic toxicity, (3) carob pod, and (4) CC_4-induced hepatic toxicity (0.5 ml/kg) plus carob pod, 50 days study duration	✓ blood AST, ALT, ALP, LDH, creatinine and urea, MDA, liver and kidney MDA, liver histological damage<br>✓ antioxidant enzymes in kidney and liver: GSH, GST, GPx, GSR, CAT	↓<br>↑
10	[84]	carob pulp flour extract (CE) obtained by optimized microwave-assisted extraction	In vivo: 42 healthy, adult, male Swiss-Webster mice, 8 weeks old, 32 - 3 g, divided into six groups (N=7/group) of (1) healthy control, (2) healthy plus paracetamol, (3) CE 100 mg/kg, (4) CE 100 mg/kg plus paracetamol, (5) CE 200 mg/kg, (6) CE 200 mg/kg plus paracetamol, 7 days study duration	✓ blood AST, ALT, creatinine, uric acid, liver MDA, expression of liver cytochrome P450 2E1 (CYP2E1), liver histological damage<br>✓ antioxidant enzymes in the liver: SOD, CAT, GPx, GST, liver glycogen storage	↓<br>↑
11	[85]	blend of carob pods and seeds extracts together with fructooligosaccharides marketed under the brand CSAT	In vivo: 36 C57/BL6J mice, 16 weeks old, divided into three groups of standard diet (N=12), high fat/high sucrose diet (N=12), high fat/high sucrose diet plus CSAT (N=12), 26 weeks study duration	✓ antioxidant capacity, blood adiponectin, interscapular brown adipose tissue, SOD-1 and GSR mRNA, vascular function, hyperemic blood flow, acetylocholine-induced aorta relaxation<br>✓ heart weight, soleus muscle weight, blood glucose, insulin, HOMA-IR, AUC, TG, T-CHOL, LDL, IL-6, macrophage infiltration and MCP-1 mRNA both in adipose tissue and skeletal muscle, IL-1 and TNF-a mRNA levels in adipose tissue, IL-6 / COX-2 / MCP-1 / TGF-β / NOX-4 / SOD-1 mRNA in arterial tissue, mRNA levels of receptors of vasoactive substances in arterial tissue, IL-6 /	↑<br>↓

				MCP-1 / TGF-β / LO / GSR / iNOS mRNA levels in myocardial tissue, arterial pressure	
12	[86]	insoluble fiber of carob rich in polyphenols (IFCP)	In vivo: 30 male New Zealand rabbits, divided in three groups (N=10/group): rabbits fed control diet, rabbits fed dyslipidemic diet plus cellulose, and rabbits fed dyslipidemic diet plus IFCP, 8 weeks study duration	✓ liver expression of sirtuin 1 (SIRT1) and peroxisome proliferator-activated receptor gamma coactivator-1alpha (PGC-1α)<br>✓ T-CHOL, TG, liver weight, hepatic steatosis, hepatic HMG-CoA, hepatic lipase, cytochrome P450, family 7, subfamily a, polypeptide 1C (CYP7A1), LDL receptor<br>✓ liver expression of glycerol phosphate acyltransferase (GPAT) and sterol regulatory element-binding protein 1C (SREBP1c)	↑<br>↑<br>↓
13	[87]	tannin-rich carob pod fiber	✓In vivo: male Sprague-Dawley rats, 28 days study duration<br>• 1ST: weighing 51 - 69 g fed on standard diet supplemented with 5% or 15% carob fiber<br>• 2ND: weighing 120 - 140 g fed on high cholesterol diet supplemented with 5% or 10% carob fiber<br>✓In vitro: in vitro adsorption of glycocholate or taurocholate	In vivo 1ST:<br>✓ BW gain, feces coprostanol / cholesterol ratio, liver weight<br>✓ bile acid excretion<br>✓ feces levels of cholesterol, coprostanol, bile acids<br>In vivo 2ND:<br>✓ excretions of campesterol, beta-sitosterol, bile acids<br>✓ liver total lipids, liver CHOL<br>In vitro:<br>✓ adsorption of bile acids	↓<br>↑<br>↔<br>↑<br>↓<br>↑
14	[88]	fiber purified, carob fruit extract (CFE)	✓In vitro: pancreatic lipase activity<br>✓In vivo: 24 male Wistar rats, divided in four groups (N=6/group) of CFE 25, CFE 50, and CFE 150 mg/kg BW and control, 1 week study duration	In vitro:<br>✓ pancreatic lipase activity<br>In vivo:<br>✓ postprandial hypertriglyceridemia and cholesterolemia<br>✓ fecal moisture, TG and CHOL	↓<br>↓<br>↑
15	[89]	natural carob fiber	In vivo: male Wistar rats, 67 ± 2 g:<br>• 1ST: three groups (N=7/group), fed on natural carob fiber (NCF), psyllium husk (PSY) and cellulose microcrystalline (CEL), study duration 7 days<br>• 2ND: six groups (N=7/group); three fed on non-supplemented cholesterol diets (groups, NCF-, PSY- and CEL) and the other three fed on cholesterol supplemented diets (groups NCF+, PSY+, CEL+), 18 days study duration	✓ BW, food efficiency, blood TG<br>✓ fecal volume<br>✓ blood T-CHOL, dietary fat absorption, dietary nitrogen absorption	↔<br>↑<br>↓

16	[90]	carob honey aqueous extract (AE) and ethyl acetate extract (EAE)	In vivo: adult male Wistar rats, 230 - 242 g, 8 days study duration:<br>• 1ST: 30 diabetic rats assigned into five groups (N=6/group) of (1) plain distilled water, (2) AE at g/kg BW, (3) EAE at 500 mg/kg BW, (4) glibenclamide, and (5) sucrose dissolved in water at 1 g/kg BW<br>• 2ND: 30 non-diabetic rats assigned into five groups (N=6 each) as above	1ST:<br>✓ blood GLU, T-CHOL, ALT, AST, ALP<br>2ND:<br>✓ blood GLU,<br>✓ T-CHOL, TG, AST, ALP	↓<br><br>↔<br>↓
17	[91]	600 mg/kg BW aqueous extract of carob pods	In vivo: 40 adult male Wistar rats, 220 - 240 g, 15 weeks old, divided into 4 groups (N=10/group): control, carob, EtOH, and EtOH plus carob, 7 days study duration	✓ blood AST, ALT, liver MDA, liver and blood H_2O_2 and iron production, hepatocellular steatosis<br>✓ antioxidant enzymes in the liver: SOD, CAT, GPx	↓<br><br>↑
18	[92]	carob seed (CS) powder adjusted to 15 % of rat food	In vivo: female Wistar albino rats, 200 - 250 g, 4 months of age, randomly divided into four groups (N=6/group) of (1) control, (2) 20% EtOH, (3) 15% CS, (4) 15% CS plus 20% EtOH, 50 days study duration	✓ blood ALT, LDH, liver MDA, liver oxidation<br>✓ antioxidant enzymes in the liver: GST, SOD, GPx	↓<br>↑
19	[98]	15% carob gum	In vivo: male adult Wistar rats, 150 - 180 g, divided into three groups (N=12/group) of control, guar gum and carob gum, 6 weeks study duration	✓ BW, blood GLU, immunoreactive Insulin, T-CHOL<br>✓ insulinemic response	↓<br>↑
20	[99]	fiber purified, carob fruit extract (CFE)	✓In vitro: α-Glucosidase activity, glucose diffusion measurement<br>✓In vivo: 24 male Wistar rats, two months old, received oral glucose load and were divided in four groups (N=6/group) of CFE 25, CFE 50, CFE 150 mg/kg BW and control, study duration 1 week	✓ inhibition of α-Glucosidase activity<br>✓ blood GLU, SGLT1 transporter, activity, glucose diffusion, postprandial glycemia, glucose AUC	↑<br>↓
21	[100]	carob fruit extract (CFE)-enriched meat (high in saturated fat and cholesterol) providing 125 mg/kg BW of CFE	In vivo: male Wistar rats, 2 months old, divided into two groups (N=8/group) of control-restructured meat, and (CFE)-enriched meat, 8 weeks study duration	✓ growth rate, dietary digestibility, blood insulin, TG, total lipids, HOMA-IR, atherogenic index, plasma arylesterase activity (AE) and AE activity-to-plasma cholesterol ratio<br>✓ QUICKI index, fecal excretion, fecal fat and moisture, AE liver activity, protein expression levels of hepatic insulin signaling pathway (InsR/PI3K/AKT/GSK3), LDL-receptor	↓<br><br><br><br><br>↑
22	[101]	carob fruit extract (CFE)-enriched meat (high in saturated fat and cholesterol) providing 125 mg/kg BW of CFE	In vivo: 24 male Wistar rats, 2 months old, divided into three different rat groups (N=8/group) of: T2DM control group, carob fruit extract-prevention group, and carob fruit extract-treatment group, 8 weeks study duration	✓ food consumption<br>✓ glycemia, liver size, liver steatosis, hepatic lipogenic transcription factors<br>✓ blood insulin, liver glycogen accumulation, pancreatic islet number and area, presence of insulin by regeneration of pancreatic beta-cells, pancreatic islets hypertrophy, protein expression	↔<br>↓<br><br>↑

				levels of hepatic insulin signaling pathway (InsR/PI3K/AKT/GSK3)	
23	[102]	carob fruit extract (CFE)-enriched meat (high in saturated fat and cholesterol) providing 125 mg/kg BW of CFE	In vivo: 24 male Wistar rats, 2 months old, divided into three different rat groups (N=8/group) of: T2DM control group, carob fruit extract-prevention group, and carob fruit extract-treatment group, 8 weeks study duration	✓ fecal excretion, fecal moisture, favorable changes in gut microbiota, fecal short chain fatty acids (acetic, propionic, butyric, isobutyric, valeric acid), mucosal thickness of the distal colon, normal structure of the lining layers of the colon<br>✓ colon paracellular permeability	↑<br><br><br><br><br>↓
24	[103]	carob fruit extract (CFE)-enriched meat (high in saturated fat and cholesterol) providing 125 mg/kg BW of CFE	In vivo: 24 male Wistar rats, 2 months old, divided into three different rat groups (N=8/group) of: T2DM control group, carob fruit extract-prevention group, and carob fruit extract-treatment group, 8 weeks study duration	✓ growth rate, fecal excretion, moisture and fat, plasma and liver arylesterase activity, LDL receptor<br>✓ dietary digestibility, glycemia, blood T-CHOL, TG, total lipids, atherogenic index, liver oxidation (VLDL-ox, TBARS, MDA), liver steatosis<br>✓ blood HDL	↑<br><br><br>↓<br><br><br>↔
25	[104]	methanolic extract from unripe carob pods	✓In vitro:<br>• 1ST: a-amylase and a-glucosidase activity<br>• 2ND: RIN-5F (rat pancreatic -cell line) and WRL68 (human hepatic cell line)<br>✓In vivo: 30 male Sprague-Dawley rats, 7-8 weeks old, injected with streptozotocin-andnicotinamide to induce T2DM, divided into five groups (N=6/group) of 500 mg/kg carob extract, 1000 mg/kg, diabetic untreated, diabetic glibenclamide-treated and healthy control, 4 weeks study duration	In vitro:<br>✓ Inhibition of a-amylase and a-glucosidase activity<br>In vivo:<br>✓ BW, blood glucose,<br>✓ blood glucose tolerance, pancreatic islet number and area, number of pancreatic beta-cells	 ↑<br><br>↓<br>↑
26	[105]	carop pulp extracts from immature and mature pods	In vivo:<br>• 1ST: Male mice (20 - 30 g) for ex vivo intestinal tests<br>• 2ND: 60 adult male Wistar rats, 200 - 250g, received alloxan to induce T2DM, divided into7 groups (N=10/group) of 50 mg/kg carob extract, 100 mg/kg carob extract, 200 mg/kg carob extract, diabetic untreated, diabetic glibenclamide-treated and healthy control, 14 days study duration	✓ dose-dependent inhibition of intestinal short-circuit current of sodium-dependent glucose transport, glucose tolerance<br>✓ blood GLU, T-CHOL, TG, creatinine, uric acid, AST, ALT	↑<br><br><br>↓
27	[106]	1.15 g/kg carob-pod pinitol preparation	In vivo: 10 male Zucker diabetic fatty (ZDF) rats, 350 g ± 30 g, 12 weeks old, divided into two groups (N=5/group) of two groups: pinitol solution or sucrose solution, 4 weeks study duration	C4A complement protein involved in the insulin secretion pathway, expression of jejunum GLUT2 transporter	↑

28	[107]	carob-derived D-pinitol powder	✓ In vitro: glucose stimulated INS-1E beta-cells<br>✓ In vivo: male Wistar rats, 4 - 5 weeks old, 400 ± 20 g, sacrificed in groups (N=5) at different times: 10, 20, 30, 60, 120 and 240 min after D-Pinitol load (100 or 500 mg/Kg)	In vitro:<br>✓ insulin secretion, pyruvate kinase, phosphorylation of the enzymes AKT and GSK-3<br>In vivo:<br>✓ no liver toxicity<br>✓ blood insulin, HOMA-IR<br>✓ blood glucagon, glucagon/insulin ratio, ghrelin	↓<br><br><br>↓<br>↑
30	[108]	methanolic extract of carob leaves	In vitro: methanol extract against glucose-mediated glycation in serum bovine albumin	acetylcholinesterase, advanced glycation end products, fructosamines	↓
31	[109]	lyophilized extracts of carob pulp and seeds	In vitro: phorbol myristate acetate (PMA)-stimulated human neutrophils	production of ROS (O^-, H_2O_2), MPO activity, p47^{phox} phosphorylation on Ser328, lactoferrin	↓
32	[110]	carob leaf extracts enriched in total oligomer flavonoids	In vitro: murine leukaemia cells (L1210)	ROS production, MDA	↓
33	[119]	Snack A: 0.1 % wakame and 5 % carob pod in oat and wheat flour dough<br>Snack B: 1 % of wakame and 5 % carob pod	In vivo: 40 male Wistar rats with MetS, 6-week-old, randomly assigned into four experimental groups (N = 10/group): (1) wheat control group, (2) oat control group, (3) snack A group, and (4) snack B group, 4 weeks study duration	✓ liver / BW ratio, liver TG and phospholipid content, serum TG, serum non-esterified fatty acids, non-HDL cholesterol levels, TBARS,<br>✓ serum HDL, AST, ALT, glucose uptake related gene and protein expression<br>✓ SOD, b-oxidation related gene expression, enzyme activity (CPT-1a, CS), lipogenic gene expression	↓<br>↔<br>↑
34	[120]	carob honey (100 mg/kg BW)	In vivo: adult male Wistar rats, randomly assigned into three experimental groups: (1) oral administration of distilled water (control group), (2), oral administration of furosemide and (3) oral administration of carob honey, 9 days study duration	✓ urine output, urine sodium and potassium<br>✓ no hypokalemia	↑

↓ lowering effect; ↑ increasing effect, ↔ no change

BW, body weight; GLU, glucose; T-CHOL, total-cholesterol; LDL, low-density lipoprotein; VLDL, very low-density lipoprotein; HDL, high-density lipoprotein; TG, triacylglycerols; CRP, C-reactive protein; HOMA-IR, homeostasis model assessment of insulin resistance; AUC, area under the curve; ACE, angiotensin converting enzyme; PGD2, prostaglandin D2; SBP, systolic blood pressure; TNF-alpha, tumor necrosis factor-alpha; IL-, interleukin; MCP-1, monocyte chemoattractant protein-1; NF-κB, nuclear factor-kappaB; TGF-β, transforming growth factor beta; GPDH, glycerol-3-phosphate dehydrogenase; ROS, reactive oxygen species; TBARS, thiobarbituric acid reactive substances; LOOH, lipid hydroperoxides; MDA, malonaldehyde; ORAC, oxygen radical absorbance capacity; CAT, catalase; GSH, glutathione; GSR, glutathione reductase; GPx, glutathione peroxidase; GST, glutathione S-transferase; SOD, superoxide dismutase; MPO, myeloperoxidase; LDH, lactate dehydrogenase; iNOS, inducible nitric oxide synthase; HMG-CoA, 3-hydroxy-3-methylglutaryl coenzyme A; GLUT2, glucose transporter 2; sodium-glucose-linked 34 transporter-1, SGLT1; ALT, alanine transaminase; AST, aspartate aminotransferase; ALP, alkaline phosphatase; T2DM, type 2 diabetes mellitus

Supplementary Table S2. Included clinical trials investigating the effects of Carob (*Ceratonia siliqua* L.) on obesity and related metabolic disorders

a/a	Ref. No	Daily Dosage	Design	Biomarker	Effect
1	[93]	A) 65 g carob snack vs. 43 g chocolate cookie snack vs. 42 g white bread vs. 25 g glucose<br>B) 40 g carob snack, 40 g chocolate cookie	randomized, single-blind, crossover design trials in healthy nonsmoking, nondiabetic individuals:<br>1ST: determination of GI, N=10;<br>2ND: effects of consuming a preload (carob or chocolate cookie), N=50 (22 men and 28 women), BMI 18 - 25 kg/m^2, age 18 - 50 years, 1wk wash out	✓ GI<br>✓ next meal's energy intake, blood GLU levels, blood GLU iAUC<br>✓ total amount of food and total carbohydrates consumed after 24h<br>✓ perceived hunger, desire to eat, preoccupation with thoughts of food, motivation to eat, thirst<br>✓ perceived fullness	↓<br>↓<br>↓<br>↓<br>↑
2	[94]	1.5 g carob seed powder in capsules (3 x 500 mg)	quasi-experimental study and clinical trial, 40 obese men, randomly assigned to four groups: (i) resistance training, (ii) carob supplementation, (iii) combined i and ii, and (v) control group, study duration 8 weeks	✓ irisin index<br>✓ improved blood lipid profile (statistical analysis not shown)	↑
3	[95]	15 g/d of a fiber rich carob pulp preparation supplemented in three different products (breakfast cereal, fruit muesli bar, powdered drink) containing 5 g of carob each	47 adult volunteers (31 women, 16 men) with serum T-CHOL 232 - 302 mg/dL, 8 weeks study duration	✓ blood T-CHOL, LDL<br>✓ blood TG, HDL<br>✓ blood GLU	↓<br>↔<br>↑
4	[96]	15 g/d of a fiber rich carob pulp preparation supplemented in bread (two servings daily) and fruitbar (one serving daily)	double blind, randomized placebo-controlled trial, 58 hypercholesterolemic patients (200 - 299 mg/dL) aged 34 - 70, assigned into two groups (N=29/group) consuming bread (two servings daily) and fruitbar (one serving daily) with or without carob pulp preparation, 6 weeks study duration	✓ blood T-CHOL, TG, LDL, apolipoprotein B:A-1 ratio<br>✓ energy intake, macronutrient intake, anthropometric values, blood glucose, HbA1C, insulin, HDL	↓<br>↔
5	[97]	8 g/d of a natural insoluble dietary fiber comprised of 80 % insoluble polyphenols from carob pod	randomized, double-blind placebo-controlled trial, 97 hypercholesterolemic patients (200 - 299 mg/dl), aged 22 - 65 years, assigned to two groups: (1) carob (N=48) and (2) placebo (N=48), 4 weeks study duration	✓ blood T-CHOL, LDL, LDL:HDL ratio, TG<br>✓ antropometric values, blood GLU, creatinine, uric acid, bilirubin, ALT, AST, ALP,	↓<br>↔
6	[111]	130 g bread with 10 % carob-seed flour	randomized, single-blind, crossover clinical trial, 10 healthy, non-smoking, non-diabetic, men and women, BMI 18 - 32, aged 18 - 50 years, participants received D-Glucose or	✓ medium to marginally high GI (≤70 on glucose scale), low GL (GL≤10 per serving)<br>✓ blood GLU blood at 15 min and 30 min, peak blood GLU, glucose iAUC, salivary insulin	 ↓

			four types of bread: white bread, coarse bran bread, fine bran and carob-seed bread	✓ fasting blood GLU	↔
7	[112]	0, 5, 10 or 20 g carob fiber rich in polyphenols	randomized single-blind cross-over study, 20 healthy adults (12 women and 8 men), BMI 18.5 - 25 kg/m^2, age 22-62 years, participants consumed 200 mL water with 50 g glucose and 0, 5, 10 or 20 g carob fiber	✓ postprandial blood GLU, insulin<br>✓ acylated ghrelin	↑<br>↔
8	[113]	carob pulp preparation rich in dietary fiber and polyphenols added in foods	randomized controlled crossover study, 19 healthy adults (9 men, 10 women) consumed standard foods with or without carob pulp (30 g)	✓ fasting acylated ghrelin, postprandial blood GLU<br>✓ postprandial TG / non-esterified fatty acids<br>✓ fasting GLU / TG / total ghrelin / non-esterified fatty acids / insulin / leptin	↑<br>↓<br>↔
9	[114]	carob tablets	seven healthy volunteers, aged between 18-55 years	glycemic index	↓
10	[106]	carob-derived pinitol-enriched beverage	Human study: randomized, double-blind, 80 volunteers (40 healthy, 40 overweight-IGT), age 18 – 72 years, BMI 19 – 40 $Kg/\ m^2$, each group assigned to two subgroups of pinitol beverage (N=20) or a matching sucrose-sweetened beverage (N=20), 6 weeks study duration	✓ blood GLU<br>✓ insulin-like growth factor acid labile subunit (IGF1BP-ALS) and complement C4A	↓<br>↑
11	[115]	carob-derived pinitol-enriched beverage (2x2.23 g/d)	randomized controlled double-blind clinical trial, 40 healthy volunteers, BMI 20 - 30 kg/m^2, age 19 - 64 years, assigned to pinitol beverage (N=20) or a matching sucrose-sweetened beverage (N=20), 12 weeks study duration	✓ blood insulin, HOMA-IR, Apo B, postprandial GLU, glucose iAUC	↓
12	[116]	carob-derived pinitol-enriched beverage	randomized, double-blind, 38 T2DM patients, age 40 - 73 years, BMI 20 - 40 $Kg/\ m^2$, assigned to two subgroups of pinitol beverage or a matching sucrose-sweetened beverage, 12 weeks study duration	✓ blood TG, HbA1c, ROS production<br>✓ endothelial function (by regulating P-selectin, rolling velocity, polymorphonuclear leucocyte adhesion)	↓<br>↑
13	[117]	carob-derived pinitol-enriched beverage	44 prediabetic patients (fasting GLU 100 - 125 mg/Dl), age 32 -72 years, BMI 20 - 40 $Kg/\ m^2$, assigned to two subgroups of pinitol beverage or a matching sucrose-sweetened beverage, 12 weeks study duration	Non-obese<br>✓ blood insulin, HOMA-IR, fasting and postprandial glucose<br>Obese<br>✓ fasting glucose, IL-6, TNF-alpha	↓<br>↓

↓ lowering effect; ↑ increasing effect, ↔ no change

BW, body weight; GLU, glucose; T-CHOL, total-cholesterol; LDL, low-density lipoprotein; HDL, high-density lipoprotein; TG, triacylglycerols; HOMA-IR, homeostasis model assessment of insulin resistance; HbA1c, Hemoglobin A1c; GI, glycemic index; GL, glycemic load; iAUC, Incremental area under the curve (iAUC); IGT, impaired glucose tolerance; ROS, reactive oxygen species; TNF-alpha, tumor necrosis factor-alpha; IL-, interleukin; ALT, alanine transaminase; AST, aspartate aminotransferase; ALP, alkaline phosphatase; T2DM, type 2 diabetes mellitus
